# Annealing Heat Treatment for Homogenizing the Microstructure and Mechanical Properties of Electron-Beam-Welded Thick Plate of Ti-6Al-4V Alloy

**DOI:** 10.3390/ma16237423

**Published:** 2023-11-29

**Authors:** Seongji Seo, Jiyong Park

**Affiliations:** 1 Advanced Joining & Additivie Manufacturing R&D Department, Korea Institute of Industrial Technology, Incheon 21999, Republic of Korea; ssg4915@kitech.re.kr; 2 Department of Materials Science and Enginering, Hanyang University, Seoul 04763, Republic of Korea; 3 Department of Convergence Manufacturing System Engineering, Korea National University of Science and Technology, Daejeon 34113, Republic of Korea

**Keywords:** Ti-6Al-4V alloy, electron beam welding, mill annealing, beta annealing, microstructure, mechanical properties

## Abstract

In the application of Ti-6Al-4V to aerospace structural components, when welding thick plates similar of the thickness of the components, microstructure and hardness gradients emerge between the base material (BM) and the joint. This leads to the issue of significant stress concentration in the BM under tensile stress. To address this problem through post-welding heat treatment, this study conducted heat treatments at temperatures both below (mill annealing, MA) and above the beta-transus temperature (beta annealing, BA) on electron-beam weldments of 18 mm thickness Ti-6Al-4V plates. Subsequently, microstructures and hardness were analyzed at different depths from the upper surface and areas (fusion zone (FZ), heat-affected zone (HAZ), and BM), and tensile properties were measured at various depths. The results indicated that α′ observed in FZ and HAZ was resolved through both MA and BA. Particularly after BA, the microstructural gradient that persisted even after MA completely disappeared, resulting in the homogenization of widmanstätten α + β. Consequently, after BA, the hardness gradient in each zone also disappeared, and the tensile strength was higher than in just-welded and MA heat-treated plates.

## 1. Introduction

Titanium (Ti) alloys are extensively used in the aerospace industry due to their high specific strength, corrosion resistance, and high-temperature properties [1,2,3,4]. To enhance productivity efficiency in aerospace component manufacturing and reduce aircraft weight, fusion welding processes can be employed for joining components [4]. Among the various fusion welding processes, the electron beam welding (EBW) method not only prevents oxidation by providing a vacuum shield but also offers the advantages of a large welding depth, small welding width and deformation, and high welding speed [5,6,7,8,9]. The application of EBW includes the joining of thick-section assemblies in structural and engine parts [7,8,9]. However, the characteristics of Ti alloy and the significant gradient of the thermal effect in EBW easily lead to strong inhomogeneity of the microstructures and mechanical properties in the welding zone: the fusion zone (FZ), heat-affected zone (HAZ), and base metal zone (BM) [4,5,6,7,8,9,10,11]. The major issue in EBW of Ti alloy is the loss of joint plasticity and toughness after welding. To obtain excellent overall characteristics in EBW joints, it is necessary to perform post-weld heat treatment (PWHT) [12].

Some researchers have conducted electron beam welding (EBW) and post-weld heat treatment (PWHT) on Ti plates with a thickness of less than 10 mm [13,14,15,16]. N.K. Babu et al. [13], C.J. Tsai et al. [14], and G. Wang et al. [15] performed annealing on EBWed plates and found that the decomposition of α’ into α + β in the fusion zone (FZ) led to a decrease in hardness and tensile strength, with most fractures occurring in the base metal (BM). Other researchers also explored EBW and PWHT on plates thicker than 10 mm [17,18,19,20,21,22,23,24,25,26,27]. W. Lu et al. [17] and J.N. Li et al. [18] conducted EBW on 50 mm and 100 mm thickness Ti plates, respectively, and observed lower hardness and strength in the top area. J. Tao et al. [22] studied the effect of micro-morphology at the fatigue crack tip in 16 mm thick welded plates annealed at 650 °C. H. Liu et al. [23,24] performed annealing on 20 mm thick EBW-ed plates and found that elongation increased after stabilization annealing. Z. Yang et al. [26] focused on the center of 60 mm thick plates that underwent EBW and solution treatment and aging (STA) heat treatment, discovering that elongation and toughness increased due to the STA heat treatment. Additionally, in the case of thick plates, most tensile fractures occurred in the BM [17,18,19,20,21,22,23,24,25,26,27].

Meanwhile, the low thermal conductivity of Ti alloys and the size effect during EBW and PWHT of thick plates result in a distribution of cooling rates inside the weldment [1,2,3]. This leads to microstructural and hardness deviations according to depths as well as zones. However, studies on PWHT of thick plates, as mentioned [19,20,21,22,23,24,25,26,27], analyze the microstructure and mechanical properties only in the middle-depth area, neglecting other depths. One of the applications of the EBW process for the Ti-6Al-4V (Ti64) alloy is in structural parts such as aft booms and spars, which are critical for resisting crack propagation [28]. However, in the case of inhomogeneously welded Ti64 in a tensile environment, strain localizes at the BM due to the hardness gradient in the joints. Consequently, if cracks appear initially in the BM, they propagate rapidly, as crack propagation is faster in a fully equiaxed or duplex microstructure than in a fully lamellar structure [3]. Therefore, to achieve greater homogenization of microstructural properties, annealing above the β-transus temperature is required for structural parts fabricated from welded Ti64.

Here, this study aimed to determine the optimal annealing heat treatment for reducing the gradient of microstructure and hardness in EBWed Ti64 plates with a thickness of 18 mm, considering various depths and zones (FZ, HAZ, and BM). The research involved a rigorous and quantitative examination of the microstructure in EBWed and post-weld heat-treated (PWHTed) joints, covering aspects such as phase transformation, grain size, and plate thickness. Additionally, micro-Vickers hardness profiles and tensile properties were measured and compared for EBWed and PWHTed conditions. The results revealed that annealing the Ti64 weldment in the β area is the most favorable heat treatment, leading to the homogenization of microstructural properties and hardness, resulting in the highest tensile strength.

## 2. Materials and Process Method

### 2.1. Materials and Electron-Beam-Welding Process

**Figure 1:**
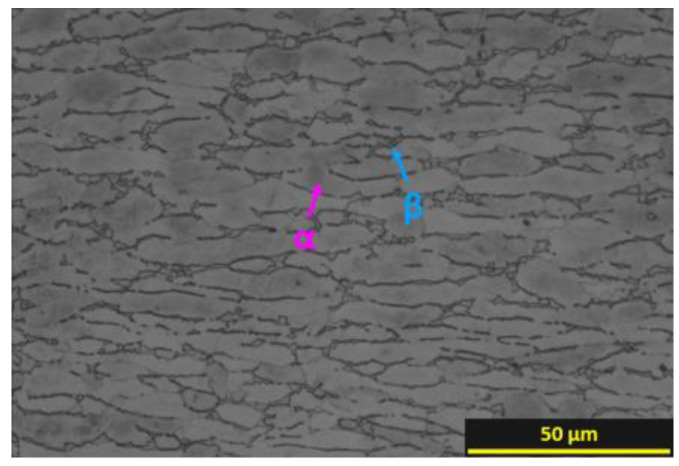
Initial microstructure of Ti-6Al-4V (Ti64) alloy used in this study.

The BM, Ti64 alloy for aerospace grade (grade 23) in this study, comprised of 18 mm thick plates (300 × 25 × 18 (mm^3^)) that were prepared by machining from the center of a 100 mm thick billet multi-forged in the α + β temperature range. The billet was provided by KPCM Co., Ltd. (Gyeongsan, Republic of Korea). The chemical composition is presented in Table 1. The microstructure exhibited a fully equiaxed structure composed of equiaxed α and β, shown in Figure 1.

**Figure 2:**
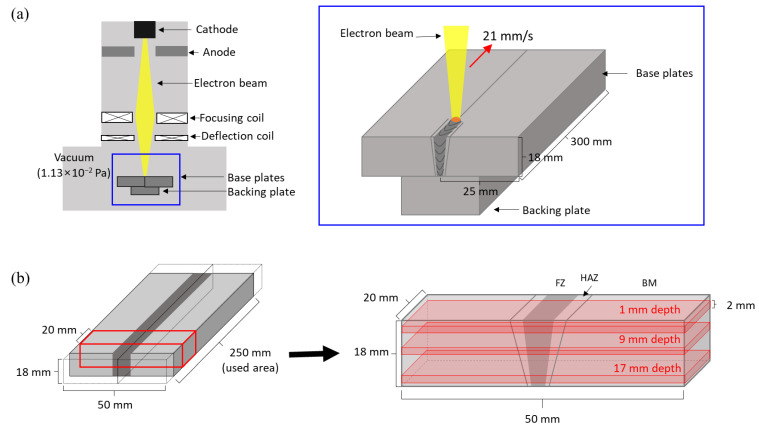
(**a**) Schematic diagram of EBW process used in this study and (**b**) marked area indicating the extracted sample for heat treatment and analysis.

This study used the EBW process, one of the fusion welding processes that utilizes an electron beam as the heat source in a high vacuum chamber, as shown in Figure 2a. Prior to clamping for the EBW process, the surface of each sample was ground and then cleaned with ethanol to eliminate any surface oxides and impurities. Base metal plates were clamped with a backing plate to prevent the flow of molten metal. Pre-heating was conducted at 250 °C, followed by the EBW process in a butt joint configuration without filler metal within a high-pressure vacuum (1.13 × 10^−2^ Pa) using the Sciaky W2000 system (Chicago, IL, USA). The operational parameters and a schematic diagram of the welding process are listed in Table 2.

### 2.2. Post-Weld Heat Treatment

The EBWed Ti64 samples were machined to dimensions of 20 × 50 × 18 (mm^3^), excluding a 25 mm section from both the starting and ending points (Figure 2b). PWHT was performed according to Table 3, using a quartz furnace chamber (<8.0 × 10^−2^ Pa). Mill annealing (MA) was conducted at 730 °C for 2 h, followed by air cooling (AC) [29]. Beta annealing (BA) was carried out at 1030 °C, which is above the β-transus temperature (998 °C), for 0.5 h, followed by air cooling [30]. Herein, the EBWed Ti64, EBW + MA heat-treated, and EBW + BA heat-treated samples are abbreviated as EBWed, MA, and BA, respectively.

### 2.3. Analysis

The EBWed, MA, and BA samples were transversely sectioned to the welding direction to analyze changes in microstructure and hardness. After sectioning, the samples were prepared for metallographic analysis involving mounting, grinding, and polishing with 1 μm diamond suspension and 0.04 μm colloidal silica suspension. Subsequently, they were etched via immersion in a solution consisting of 10% HF, 5% HNO_3_, and 85% H_2_O.

X-ray diffraction (XRD, Xpert-PRO MPD, PANalytical) analysis was performed for the FZ and BM area at the middle depth in a 2 h range of 30–90° at a scan rate of 0.02 min (X-ray: Cu Ka). Macro and microstructural observations were conducted for each zone (BM, HAZ, and FZ) and depth (1, 9, and 17 mm) using both an optical microscope (OM, HRM-300, Huvitz, Houston, TX, USA) and a scanning electron microscope (SEM, NNS-450, FEI, Hilsboro, OR, USA). The average grain size in each area was calculated using I-solution DT software ver. 26.5 (IMT i-solution Inc., Burnaby, BC, Canada), following the planimetric method outlined in the ASTM E112-13 standard [31]. The thickness of α and/or α′ plates was determined from SEM images, with the average value based on measurements from three plates in five areas.

**Figure 3:**
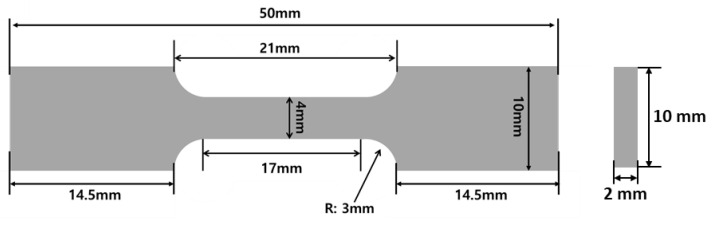
Tensile sample for used in this study.

Hardness profiles of EBWed, MA, and BA were measured in both directions from the center of the FZ to BM, averaged at each point, and measured at various depths (1, 9, and 17 mm) using a micro-Vickers hardness tester (HM-210 B, Mitutoyo, Kawasaki, Japan). The testing was conducted at a load of 500 g, a dwell time of 12 s, and an interval of 0.25 mm. Tensile strength and elongation were also measured using a universal testing machine (5982, Instron, Norwood, MA, USA) at room temperature. The testing speed was 0.001/s, and the samples were extracted at depths of 1, 9, and 17 mm, with a gauge length of 17 mm and a width of 4 mm, where FZ was in the middle of the gauge length (Figure 3). Fractographies were analyzed by using SEM.

## 3. Results and Discussion

### 3.1. Microstructure Evolution

#### 3.1.1. Macrostructure

**Figure 4:**
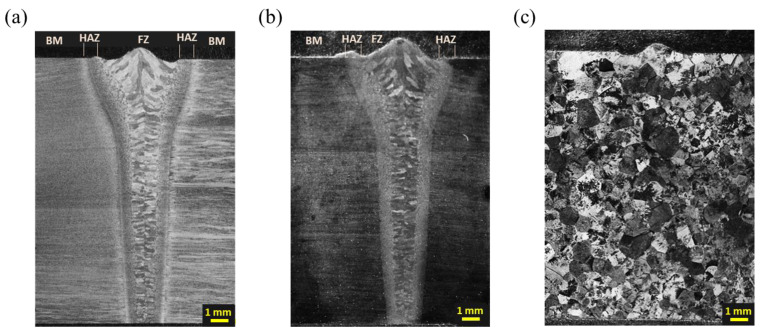
Cross-sectional macrostructural images of (**a**) EBWed, (**b**) MA, and (**c**) BA.

Figure 4 shows the cross-sectional macrostructures of the EBWed, MA, and BA samples. All the samples exhibited undercut owing to surface evaporation and expulsion of the molten materials during the welding process [25,32]. The undercut depth ranged from 0.12 to 0.25 mm, which was below the allowable undercut depth of 7% of the sheet thickness specified in ‘AWS D17-1 Specification for Fusion Welding’ [33].

As illustrated in Figure 4a, EBWed Ti64 can be divided into three zones: FZ, HAZ, and BM. The FZ is characterized by transformed β and its columnar phase, growing from the fusion line to the weld center. The size of transformed beta grains in the FZ decreases as the depth increases from the upper surface. In Figure 4b, it can be seen that even though MA heat treatment was performed after welding, the divided areas (FZ, HAZ, BM) that existed after welding still remained. However, in Figure 4c, the BA sample exhibits the fully same microstructure, which consists of transformed β, because the holding temperature is above the β-transus temperature. Details regarding the microstructures for each zone and depth of all the samples are provided in Section 3.1.2.

#### 3.1.2. Microstructure

**Figure 5:**
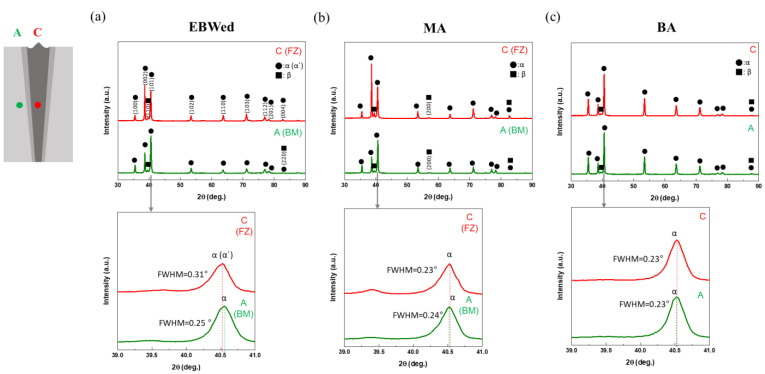
XRD patterns of A and C zones of the center depth from the upper surface in EBWed and PWHTed Ti64: (**a**) EBWed Ti64, (**b**) MA, and (**c**) BA.

Before analyzing the microstructure of each area in depth and zone with OM and SEM, XRD was used to identify the phases present in the BM (A zone) and FZ (C zone) at the mid-depth point of EBWed, MA, and BA (Figure 5). In Figure 5a, the BM area, similar to the as-received Ti64, exhibits α (ICSD 98-009-9778) and β phase (ICSD 98-007-6165) peaks. Regarding the β phase in the A zone of Figure 5a, only (100) and (220) β peaks are detected. In the C area (FZ) of EBWed, the (220) β peak is absent; this is because rapid cooling leads to the retention of a small amount of β that is not transformed to α′. The magnified range of 39–41° shows a shifted and broadened α peak in the FZ, providing evidence of the presence of α′ [34,35]. In Figure 5b, for the MA sample, there are no shifted or broadened α peaks, and (200) β peaks are additionally detected. This indicates that during MA, some of β phase occurrences take place. After BA, as shown in Figure 5c, all the peaks correspond to α and β phases. The α peak in the 39–41° range has almost the same full width at half maximum (FWHM) and detected area for both A and C zones.

**Figure 6:**
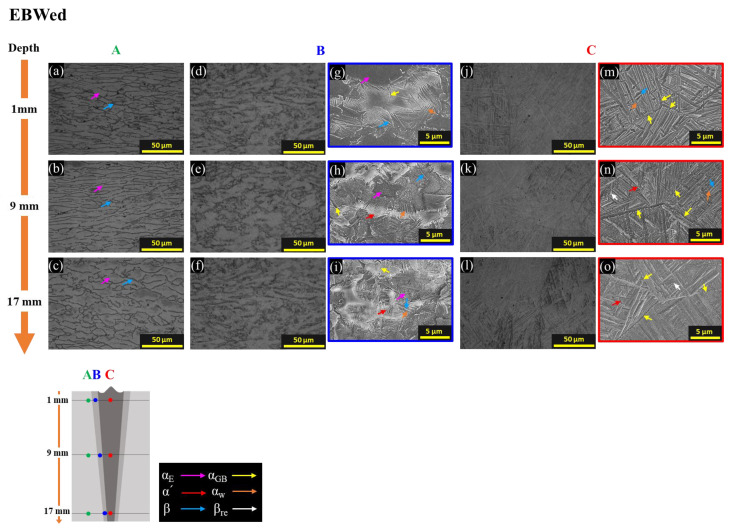
OM and SEM images for each zone and depth of EBWed with colored arrows that are pink for equiaxed α (α_E_), yellow for grain boundary α (α_GB_), red for α′, orange for widmanstätten α plate (α_w_), blue for β phase, and white for retained β (β_re_); (**a**–**c**) OM of zone A (BM), (**d**–**f**) OM and (**g**–**i**) SEM of zone B (HAZ), (**j**–**l**) OM and (**m**–**o**) SEM of zone C (FZ).

**Figure 7:**
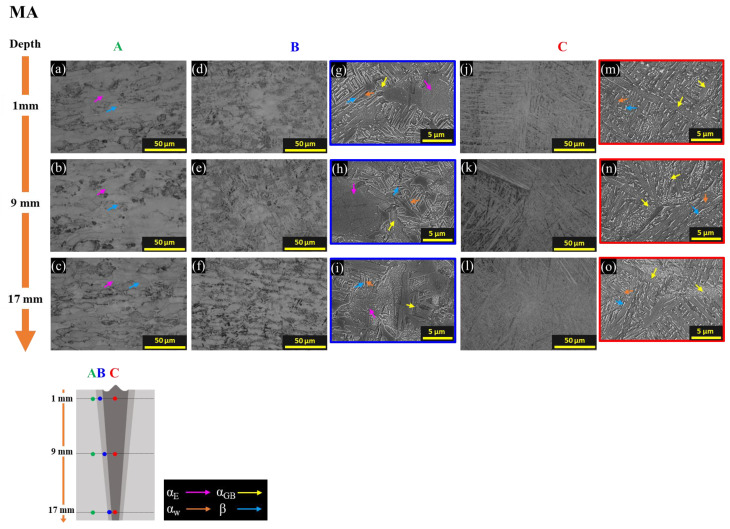
OM and SEM images for each zone and depth of MA with colored arrows that are pink for α_E_, yellow for α_GB_, orange for α_w_, and blue for β; (**a**–**c**) OM of zone A, (**d**–**f**) OM and (**g**–**i**) SEM of zone B, (**j**–**l**) OM and (**m**–**o**) SEM of zone C.

**Figure 8:**
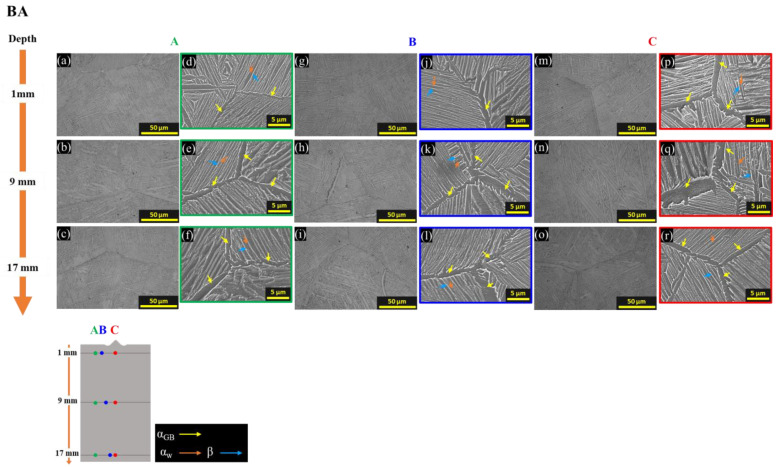
OM and SEM images at the center for the depths of BA with colored arrows that are yellow for α_GB_, orange for α_w_, and blue for β; (**a**–**f**) OM and SEM of zone A, (**g**–**l**) OM and SEM of zone B, and (**m**–**r**) OM and SEM of zone C.

Figure 6 and Figure 7 present microstructures for each zone and depth of EBWed and MA. Observation areas are indicated by arrows and colors: A in green, B in blue, and C in red, representing the BM, HAZ, and FZ, respectively. Figure 8 illustrates microstructures of the BA sample for A to C areas. Observation depths range between 1, 9, and 17 mm from the upper surface to the bottom of the samples. Colored arrows represent the following: pink for equiaxed α (α_E_), yellow for grain boundary α (α_GB_), red for α′, orange for widmanstätten α plate (α_w_) in transformed β, blue for the β phase, and white for retained β (β_re_).

Figure 6a–c are the BM (zone A) in the EBWed, comprising α_E_ and β. The HAZ (zone B), displayed in Figure 6d–i, exhibits a duplex microstructure composed of α_E_, transformed β, and some α_GB_. In this case, the transformed β forms along the boundaries of α_E_. Similar findings of an as-welded microstructure have been reported by other researchers [19,20,21]. This is because, during the heating of EBW, the grain boundaries of α_E_ serve as starting points for the transformation from α to β. Subsequently, during the cooling phase of EBW, β is transformed into α_w_ + β and/or α′. Figure 6j–o represent the FZ (zone C) during welding. Here, the metal melted upon crossing the liquidus temperature and solidified rapidly, resulting in the presence of fully transformed β and α_GB_. Since the top surface is closer to the heat source, the cooling rate at a depth of 1 mm is slower, leading to the presence of α_w_ + β (Figure 6j). At a depth of 17 mm (Figure 6o), the transformation of α′ occurred due to rapid cooling, even though this area is in contact with the backing plate. Additionally, there is some retained β (β_re_), which is the non-transformed phase of β, due to cooling being too rapid for transformation to α′. Therefore, because cooling rate distribution across different depths in zone B exhibits a similar trend to zone C, the amount of α′ in the duplex microstructure increases as the depth increases from 1 to 17 mm, which is far from the top surface, as shown in Figure 6g–i.

During MA, some of dislocations in the BM were resolved and recrystallized [1,2,3], so a microstructure in zone A is similar to that of EBWed, which consists of α_E_ and β (Figure 7a–c). According to the results of JMatPro calculations, at 730 °C, the α and β phases are present in approximately 89.4% and 10.6%, respectively [36], so during MA, there is transformation of α′ into equilibrium α_w_ and β phases. Therefore, microstructures of HAZ, in zone B, consist of some α_GB_, α_E_, α_w_, and β, as shown in Figure 7d–i. In the case of FZ, in zone C, Figure 7j–o exhibit a fully α_w_ + β structure.

Figure 8 shows the microstructures of BA. All zones (A to C) and all depths (1, 9, and 17 mm positions from the top surface) exhibit the same microstructure, characterized by transformed β phases consisting of the α_w_ + β structure (Figure 8a–r). The occurrence of this uniform microstructure over the entire range is because during BA heat treatment, it was 100% transformed into the β phase just before cooling, and then during air cooling, the phase transformation occurred to the α_w_ + β structure. The thickness of α_w_ plates and α_GB_ is coarser at a 9 mm depth because of the lower cooling rate at the center during air cooling of the BA heat treatment.

**Figure 9:**
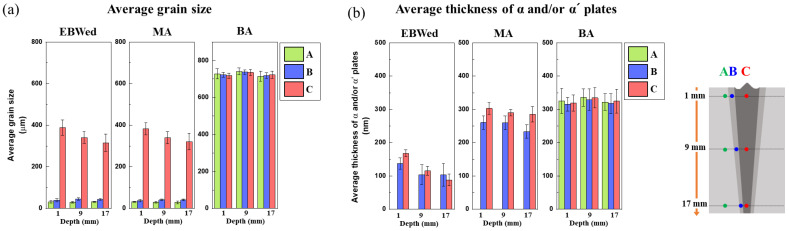
Quantitative measurement results of microstructural characteristics for EBWed, MA, and BA according to zones and depths; (**a**) average grain size, (**b**) average thickness of α and/or α′ plates.

Figure 9 presents the results of a quantification analysis of grain size and the thickness of α and/or α′ plates, calculated from Figure 4, Figure 6, Figure 7 and Figure 8. The average values are calculated for zones A, B, and C at depths ranging between 1, 9 and 17 mm. The grain size was measured and averaged at three locations in each zone and depth. The thicknesses of α plates for EBWed and MA were observed in zones B and C because the BM exhibits a fully equiaxed α and β microstructure.

As shown in Figure 4a,b, zone C of both EBWed and MA exhibits a larger prior β grain size compared to the HAZ and BM areas, and when increasing the depths from the top surface in the FZ, a smaller grain size exists due to a lower thermal effect from the electron beam. However, in Figure 4c for BA, the prior beta grain size is larger than in the other samples, and it is larger at the center depth than at the surface. Therefore, as shown in Figure 9a, all zones of both EBWed and MA exhibit similar values, with zone C being approximately 900 to 1100% larger than zones A and B. Additionally, the grain size varies according to depth, with the 17 mm depth area being smaller by about 18~23% compared to the 1 mm depth. In the BA sample, the grain size exceeds 700 μm, and at a depth of 9 mm, it is larger than in the other areas.

In Figure 9b, the thickness of the α and/or α′ plates in the EBWed shows a decreasing trend in plate thickness from 1 to 17 mm depths due to the amount of α′. Therefore, the 17 mm depth area has plates that are 25 to 50% finer than the 1 mm depth, and zone C at 17 mm depth exhibits a smaller value than zone B, primarily due to the amount of α′. After MA, the plate thickness is larger than in EBWed due to the α + β transformation and growth of α plates. Thus, in Figure 9b, the MA sample has coarser plates than EBWed by about 90~200%, and the gradient according to depths decreases. The thickness of α plates in BA is similar to that in MA, and at the 9 mm depth of BA, it has a slightly higher value than the others. However, the differences between all the zones and depths in BA are insignificant.

**Figure 10:**
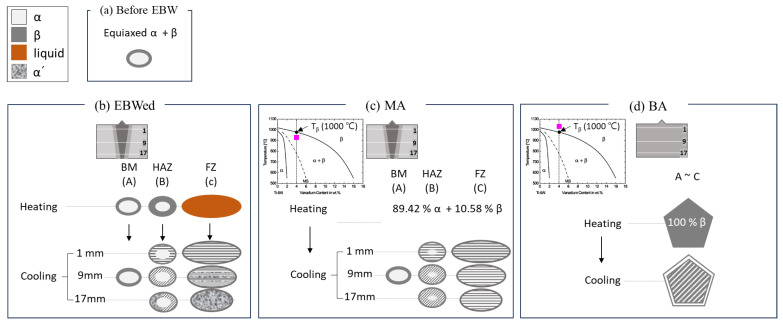
Schematic diagram of metallurgical mechanism during EBW and PWHT of Ti64 in this study; (**a**) before EBWed Ti64, (**b**) EBWed, (**c**) MA, and (**d**) BA.

A schematic diagram illustrating microstructure changes according to phase transformation by area and depth during EBW and PWHT is presented in Figure 10. In the case of EBWed Ti64, which had a fully equiaxed structure before welding (Figure 10a), FZ has a fully α′ and/or α_w_ + β structure within coarse prior β grains formed through the cooling of the molten alloy during welding (Figure 10b). In the FZ of Figure 10b, the 1 mm depth from the upper surface, close to the heat source, exhibits an α_w_ + β structure due to slower cooling, while the 17 mm depth area reveals a fully α′ structure. In the case of the HAZ, the partial area was heated, and after cooling, β transformed into α′ and/or α_w_ + β. Therefore, HAZ has a duplex structure, consisting of equiaxed α and transformed β phases (α′ and/orα_w_ + β). After MA, the α′ transforms intoα_w_ + β, and the α plates already formed after welding become coarser (Figure 10c). However, even after MA, macrostructural differences between each zone (BM, HAZ, FZ) distinguished in EBWed were still observed. In the case of BA in Figure 10d, just before cooling, it consisted of a single β phase with a grain size exceeding 700 μm in the whole area, but during cooling, β was transformed into α_w_ + β. Therefore, after BA, the distinction between each zone disappears and the entire area can be considered as one.

### 3.2. Mechanical Property

#### 3.2.1. Hardness Profile

**Figure 11:**
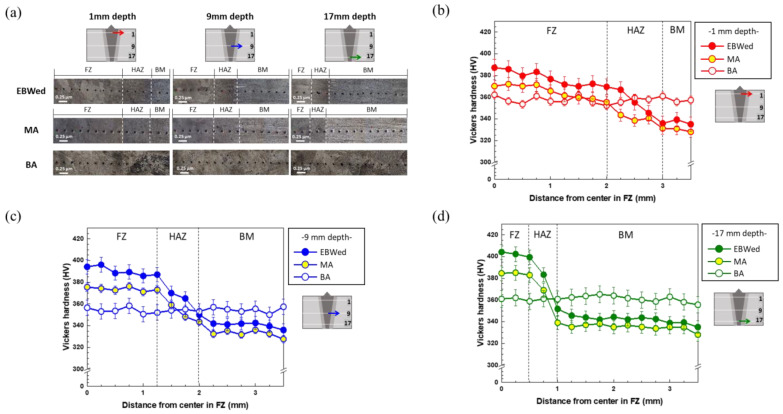
Micro-Vickers hardness profiles and measurement areas of EBWed, MA, and BA according to each zone and thickness; (**a**) hardness measurement areas, (**b**) 1 mm depth, (**c**) 9 mm depth, and (**d**) 17 mm depth.

Figure 11 presents the micro-Vickers hardness profile from the center of the FZ to the BM area of EBWed, MA, and BA according to the depths (1, 9, 17 mm) from the upper surface. The divided zones from FZ to BM are determined based on the actually measured points, as shown in Figure 11a. In EBWed and MA, the FZ exhibits higher hardness values than the HAZ and BM areas due to its microstructure characteristics, where the FZ has α′ and/or α_w_ plates compared to the HAZ area. The length of the FZ is shorter from 1 mm to 17 mm depth (Figure 4), and this tendency is reflected in the hardness profile. However, in BA, all values are similar across each zone and depth due to the uniform α_w_ + β structure and there are similar plate thicknesses throughout the entire area.

**Figure 12:**
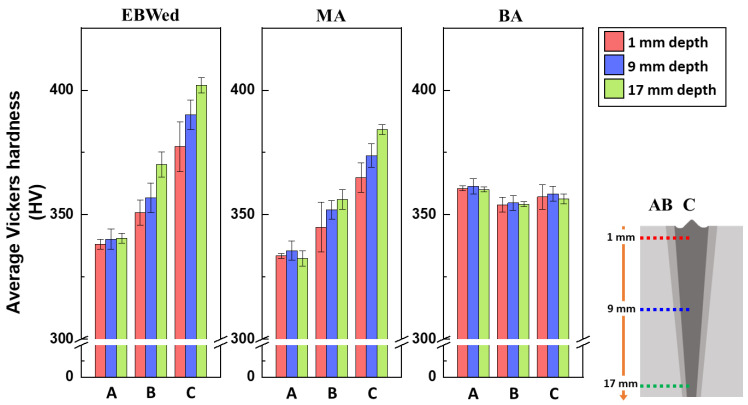
Average hardness values of each zone in EBWed, MA, and BA according to depth.

Figure 12 illustrates the average hardness values in each zone (A, B, and C) at different depths (1, 9, and 17 mm). In the case of EBWed, the C and B zones exhibit higher hardness values than A because α′ and/or α_w_ plates are present in B and C, and the size of the α plates is smaller than the α_E_ size, a phase that mainly exists in zone A. Additionally, the 17 mm depth area consists of a greater amount of finer α′ than at 1 mm, resulting in higher hardness values of B and C in the 17 mm depth, by about 7%. The hardness of MA is smaller than EBWed because α′ was transformed into α_w_ plates and the plates grew. The gradient according to depths is slightly decreased due to smaller differences in the plate thickness at each depth. BA has almost the same hardness from A to C and from 1 mm to 17 mm depths. The values in B and C of BA are lower than EBWed and MA because the thickness ofα_w_ plates is coarser than in the other samples. Meanwhile, the average hardness value of the A zone in BA is higher than in EBWed and MA. This is because the A zone in EBWed and MA mainly consists of 30 μm of α_E_, but BA mainly consists of α plates of 300~350 nm. That means the A zone in BA has a larger amount of α/β interfacial area than EBWed and MA. Therefore, more α/β interfacial area prevents dislocation movement, which means that A in BA has much higher hardness than EBWed and MA.

Concerning the indentation size, Table 4 presents the average horizontal and vertical lengths of indentations in the FZ (C zone) and BM (A zone) at 1 mm to 17 mm depths. X. Wang et al. [37] investigated why resistance to deformation is higher at smaller indentation sizes. In this study, deformation of the 17 mm depth in the FZ of EBWed was the most difficult due to the hard and fine α′ plates, so the highest hardness value was measured in that area. In contrast, the BM in MA could be easily deformed due to the smaller amount of interfacial area, and relieved stress and dislocations during annealing.

Furthermore, the relationship between hardness and toughness is inversely proportional [38]. Therefore, the FZ of EBW reveals the greatest hardness, and so it can be expected that without PWHT, the joint has the least toughness. In contrast, the best joint toughness can be expected to be obtained after BA.

#### 3.2.2. Tensile Properties

**Figure 13:**
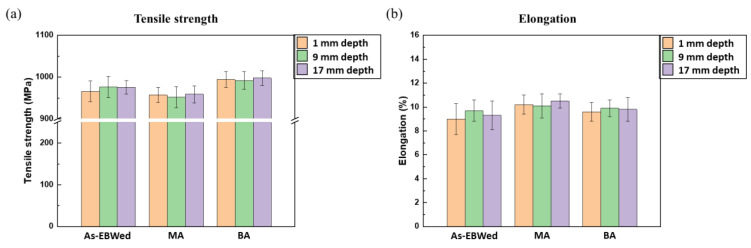
Tensile properties of EBWed, MA, and BA according to depth; (**a**) tensile strength and (**b**) elongation.

**Figure 14:**
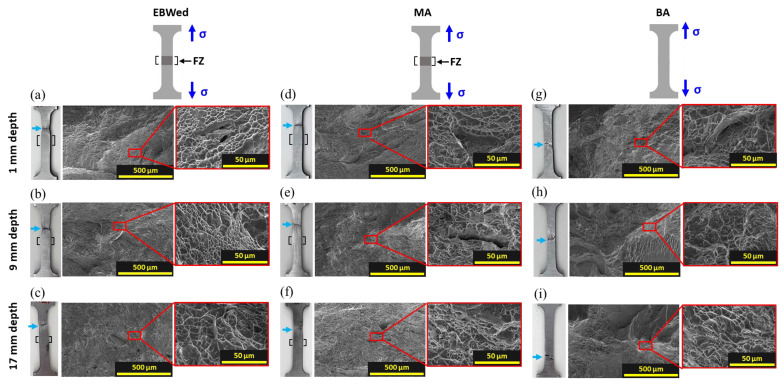
Tensile fractured samples and fractographies of EBWed, MA, and BA according to depths; (**a**–**c**) EBWed, (**d**–**f**) MA, and (**g**–**i**) BA.

Figure 13 and Table 5 present tensile strength and elongation values at 1 to 17 mm depths from the upper surface for EBWed, MA, and BA. The average tensile strength is decreased after MA, but that is increased after BA. Meanwile, average elongation is slightly increased after both heat treatments. In EBWed and MA, fractures occurred at the BM, whereas in BA, fractures occurred within the gauge length (Figure 14a–i).

In a study by A.S.H. Kabir et al. [39], during tensile testing of welded and stress-relieved heat-treated Ti64, strain was localized and fractures occurred at the BM that had a fully equiaxed microstructure because finer α′ plates in the FZ area led to an increase in resistance to deformation when compared with α_E_. Similarly, in Figure 14a–f, it can be seen that in EBWed and MA, fracturing (sky blue colored arrow) was in the BM and plastic deformation was observed only in that zone, not the entire area. The fractographies of Figure 14a–f had various sizes and shallow dimples, and some cracks existed around the torn structure [40,41,42]. However, in the case of BA, which had a homogeneous microstructure, in Figure 14g–i, it can be seen that the strain was relatively uniform, with no large, localized deformation within the gauge lengths. In the fractography of BA, trans-granular fracturing was observed with elongated dimples arranged in the grain [43]. This means the deformation in BA weldments occurs uniformly along α plates, which have similar thicknesses and is mainly composed. Consequently, in EBW and MA, stress was concentrated in the BM area, mostly composed of an α_E_ of 30 μm, leading to deformation occurring only at that location. But in the case of BA, deformation was relatively uniform due to its homogeneous microstructure, and because it is composed of 300~350 nm α_w_ plates, BA, which is finer and has more interfaces, has higher strength.

However, the deviation in tensile properties according to depths is insignificant for all the samples. Because there is almost no microstructural difference in each fractured sample according to depth, both EBWed and MA exhibit a fully equiaxed microstructure in the BM zone, and BA has the same microstructure throughout.

According to Pang, J.C., et al. [44] and Huang L.J., et al. [45], fatigue strength is proportional to tensile strength. Therefore, achieving microstructural and hardness homogenization across different zones and depths after BA of EBWed Ti64 is expected to result in higher fatigue strength compared to the solely EBWed or MA-treated ones.

## 4. Conclusions

The present study has investigated the microstructure and mechanical property distributions of EBWed and PWHTed Ti64 (MA and BA), considering each zone and depth within the weldment. The main conclusions can be summarized as follows:

1. In EBWed thick plates, the FZ, HAZ, and BM zones are divided. FZ is shown to comprise α′ and/or widmanstätten α and β, and α′ is dominant in areas far from the upper surface of the FZ. After MA, α′ in EBWed sample is transformed to widmanstätten α and β, but the divided zones still remain. Meanwhile, BA sees microstructural homogenization to fully widmanstätten α and β in transformed β, so there are no divided zones or gradients of phases over the entire area, and the microstructural characteristics at each depth are insignificant compared to solely EBWed and MA.
2. The Micro-Vickers hardness of EBWed is increased from BM to FZ, as well as far from the upper surface. After MA, the gradients of the hardness profile according to each zone and depth are smaller than for EBWed. However, after BA, the hardness values are similar across the entire area, so there are insignificant gradients. The BA heat-treated sample has a higher tensile strength than the others.

## Figures and Tables

**Table 1:** Chemical composition of Ti64 alloy used in this study (wt.%).

Ti	C	Al	V	Fe	O	H	N
Bal.	0.005	6.25	4.1	0.21	0.11	<0.0003	0.004

**Table 2:** Electron beam welding (EBW) parameters.

Voltage (kV)	Current (mA)	Power (kW)	Speed (mm/s)	Vacuum Level (Pa)
54	150	8.1	21	1.13 × 10^−2^

**Table 3:** Post-weld heat treatment (PWHT) conditions for this study.

	Temperature (°C)	Time (h)	Cooling Condition
Mill annealing (MA)	730	2	AC
β annealing (BA)	1030	0.5	AC

**Table 4:** Horizontal and vertical lengths of indentations in terms of average values of FZ and BM at each depth of EBW, MA, and BA.

	Depth (mm)	FZ (C Zone)		BM (A Zone)	
		Horizontal (μm)	Vertical (μm)	Horizontal (μm)	Vertical (μm)
EBWed	1	49.83	50.59	53.57	53.45
	9	49.45	50.21	53.76	53.76
	17	48.26	48.11	54.02	53.38
MA	1	51.73	51.92	54.00	55.69
	9	51.23	51.86	53.13	54.69
	17	50.59	50.72	55.28	54.86
BA	1	51.51	51.12	50.48	50.07
	9	51.80	52.50	51.92	52.50
	17	50.53	51.10	50.51	51.16

**Table 5:** Tensile properties of EBWed, MA, and BA samples according to depth.

	Tensile Strength (MPa)			Elongation (%)		
	EBWed	MA	BA	EBWed	MA	BA
1 mm depth	965.82	957.25	994.0	9.0	10.2	9.6
9 mm depth	976.24	952.0	992.07	9.7	10.1	9.9
17 mm depth	975.15	958.65	997.50	9.3	10.5	9.8

## Data Availability

Data are contained within the article.
